# Development and Evaluation of an Injectable Chitosan/β-Glycerophosphate Paste as a Local Antibiotic Delivery System for Trauma Care

**DOI:** 10.3390/jfb9040056

**Published:** 2018-10-12

**Authors:** Logan Boles, Christopher Alexander, Leslie Pace, Warren Haggard, Joel Bumgardner, Jessica Jennings

**Affiliations:** Department of Biomedical Engineering, University of Memphis, Memphis, TN 38152, USA; lrboles@memphis.edu (L.B.); cmlxndr2@memphis.edu (C.A.); lrpace@memphis.edu (L.P.); whaggrd1@memphis.edu (W.H.); jbmgrdnr@memphis.edu (J.B.)

**Keywords:** chitosan, local antibiotic delivery, biofilm, complex musculoskeletal wounds, trauma care, injectable, in vivo evaluation, thermo-paste

## Abstract

Complex open musculoskeletal wounds are a leading cause of morbidity worldwide, partially due to a high risk of bacterial contamination. Local delivery systems may be used as adjunctive therapies to prevent infection, but they may be nondegradable, possess inadequate wound coverage, or migrate from the wound site. To address this issue, a thermo-responsive, injectable chitosan paste was fabricated by incorporating beta-glycerophosphate. The efficacy of thermo-paste as an adjunctive infection prevention tool was evaluated in terms of cytocompatibility, degradation, antibacterial, injectability, and inflammation properties. In vitro studies demonstrated thermo-paste may be loaded with amikacin and vancomycin and release inhibitory levels for at least 3 days. Further, approximately 60% of thermo-paste was enzymatically degraded within 7 days in vitro. The viability of cells exposed to thermo-paste exceeded ISO 10993-5 standards with approximately 73% relative viability of a control chitosan sponge. The ejection force of thermo-paste, approximately 20 N, was lower than previously studied paste formulations and within relevant clinical ejection force ranges. An in vivo murine biocompatibility study demonstrated that thermo-paste induced minimal inflammation after implantation for 7 days, similar to previously developed chitosan pastes. Results from these preliminary preclinical studies indicate that thermo-paste shows promise for further development as an antibiotic delivery system for infection prevention.

## 1. Introduction

High-energy trauma to the musculoskeletal system can cause complex open wounds characterized by lacerations, abrasions, and/or open fractures [1,2]. Complex musculoskeletal wounds, especially open wounds, are highly susceptible to bacterial contamination, with infection rates ranging from 20% to 50% [3,4]. Necrotic tissue surrounding the wound suffers from diminished perfusion, effectively restricting leukocytes and oral or IV administered antibiotics from accessing the site of injury. The wound provides a sheltered anaerobic environment that is favorable for bacterial proliferation [2]. Further, the complex morphology of severe musculoskeletal wounds creates a large surface area for bacteria to adhere, the initiating mechanism in biofilm formation, which can be resistant to antibiotics [5,6]. Biofilm-forming bacteria, such as *Staphylococcus aureus* and *Pseudomonas aeruginosa*, are commonly present in complex open musculoskeletal infections [7]. Some biofilm-containing infections can be eradicated with antibiotics administered systemically, but the dangerously high concentrations may produce nephrotoxicity or ototoxicity, among other ailments [8]. Local delivery systems used as adjunctive therapy to standard prophylactic antibiotics may be used to achieve high levels of antibiotic capable of eliminating biofilm-associated bacteria in local tissue without systemic concentrations reaching levels that produce toxic adverse effects [3,9].

Local delivery of antibiotics from biomaterial systems supplements systemic therapy by increasing antibiotic concentrations at the local site of contamination in complex wounds. Current available local delivery systems for complex musculoskeletal trauma applications including poly(methyl methacrylate) (PMMA), calcium sulfate (CaSO_4_), and collagen have significant shortcomings [8,10]. PMMA possesses excellent mechanical properties, but it requires a secondary surgery for removal from the body, may serve as a nidus for secondary infection, and is incompatible with heat-labile antibiotics [11]. Ability to be resorbed by the body and providing calcium to facilitate fracture healing are assets of CaSO_4_, but inadequate wound coverage and rapid resorption resulting in serous wound drainage are undesirable characteristics [3,12]. Biodegradable collagen local delivery systems have excellent biocompatibility; however, the rapid release of antibiotics does not match its degradation rate, and wound coverage does not meet clinical needs [8].

Chitosan, an abundant polysaccharide, has been investigated for local antibiotic delivery due to its biocompatibility, biodegradation to non-toxic degradation products, and tailorability of degradation and drug delivery rates through alteration of molecular weight, degree of deacetylation (DDA), or cross-linking [13,14,15,16]. Lyophilized chitosan sponges were developed and are capable of local therapeutic antibiotic release, but they have limited ability to conform to complex wound morphologies, as well as an unwanted initial burst release of loaded antibiotics [17,18,19]. To address the limitations of chitosan sponges, an injectable paste of finely ground, lyophilized chitosan hydrated with antibiotic solution has been developed and has shown potential as an adjunctive infection-prevention tool [20,21]. The chitosan paste eluted inhibitory levels of antibiotics for 3 days, degraded in a similar time frame, and improved wound coverage. However, significant injection force made application of the paste difficult, and migration from the injection site could reduce efficacy. Thus, there is a need to improve the chitosan paste by reducing the force needed for injection and to prevent migration of the paste from the wound bed.

**Figure 1:**
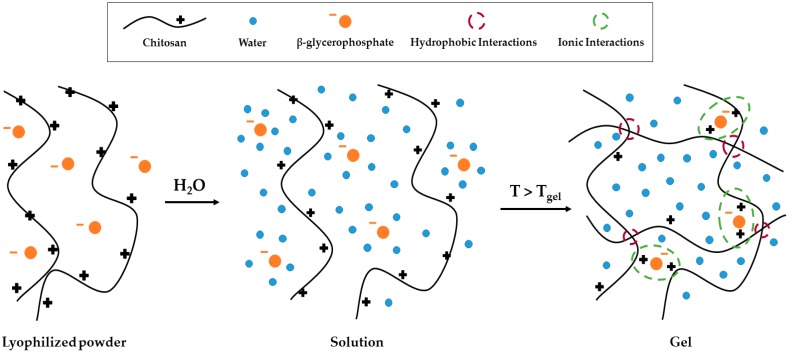
Schematic of proposed gelation of beta-glycerophosphate (β-GP) and chitosan thermo-paste delivery system.

Beta-glycerophosphate (β-GP) forms a polyelectrolyte complex with chitosan that has low viscosity at low temperatures and gels at higher/body temperatures, as shown in Figure 1 [22,23]. Taking advantage of this thermo-gelling characteristic, the addition of β-GP to chitosan paste may reduce the viscosity of the paste at room temperature to improve its injectability and wound coverage. Then, gelation would occur at normal body temperature to form a thick paste that would not migrate from the wound site and would help maintain high local antibiotic concentrations at the local injury site to prevent infection.

The goal of the current study is to develop an adjunctive infection prevention therapy for trauma care. It is hypothesized that by modifying the chitosan paste to include β-glycerophosphate, a thermo-responsive paste will be formed that is easily injectable, provides effective wound coverage, is capable of point-of-care loading with antibiotic solutions, locally delivers therapeutic levels of antibiotics, and degrades over the course of fracture healing. Specific objectives of this study were to determine the cytocompatibility, antibiotic release characteristics, enzymatic degradation, injectability, and in vivo tissue inflammatory response of the thermo-paste.

## 2. Results

### 2.1. Cytocompatibility

**Figure 2:**
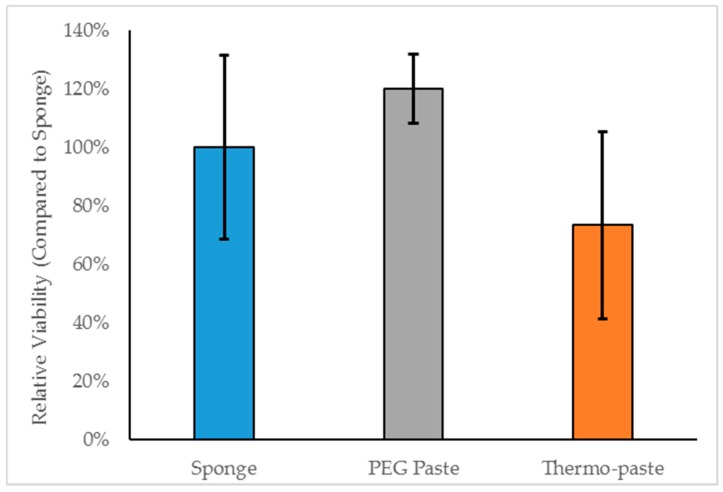
Relative viability of NIH3T3 cells after 72 h of indirect exposure to chitosan samples (n = 3); data is presented as mean ± standard deviation (STD).

The cytocompatibility of thermo-paste met the cytocompatibility standard outlined in ISO 10993-5 [24]. No statistically significant differences in relative viability were observed between the groups at the 0.05 level of significance (Figure 2).

### 2.2. Antibiotic Elution and Activity

**Figure 3:**
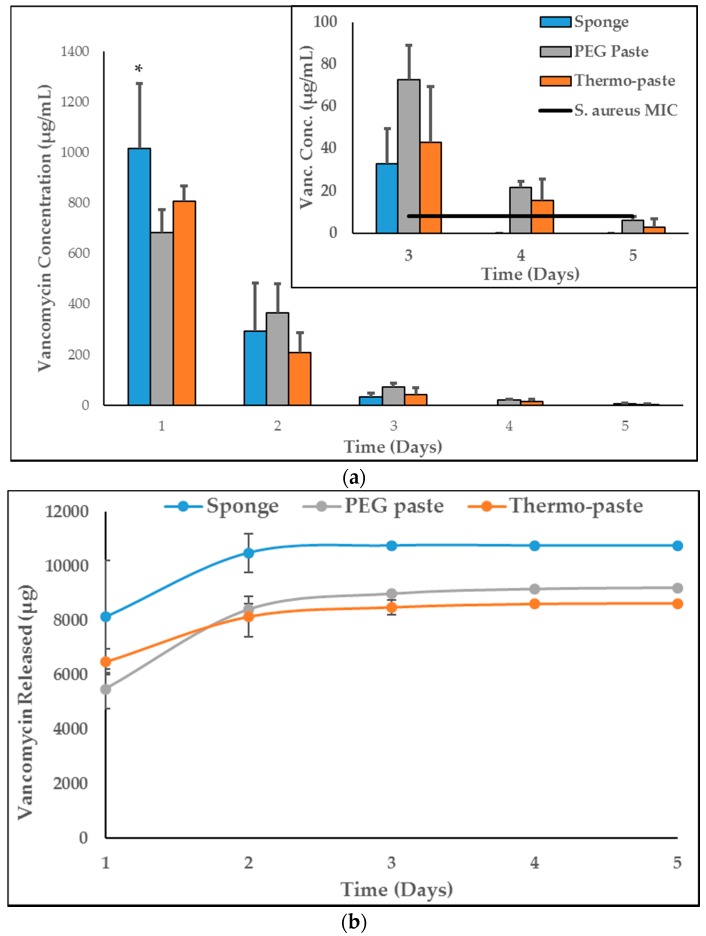
(**a**) Concentrations of vancomycin in eluate samples, (**b**) cumulative vancomycin released (n = 3). Data presented as mean ± STD. Minimum inhibitory concentration (MIC) values reported are lab generated values. * represents a significant difference compared to PEG paste (*p* < 0.05).

**Figure 4:**
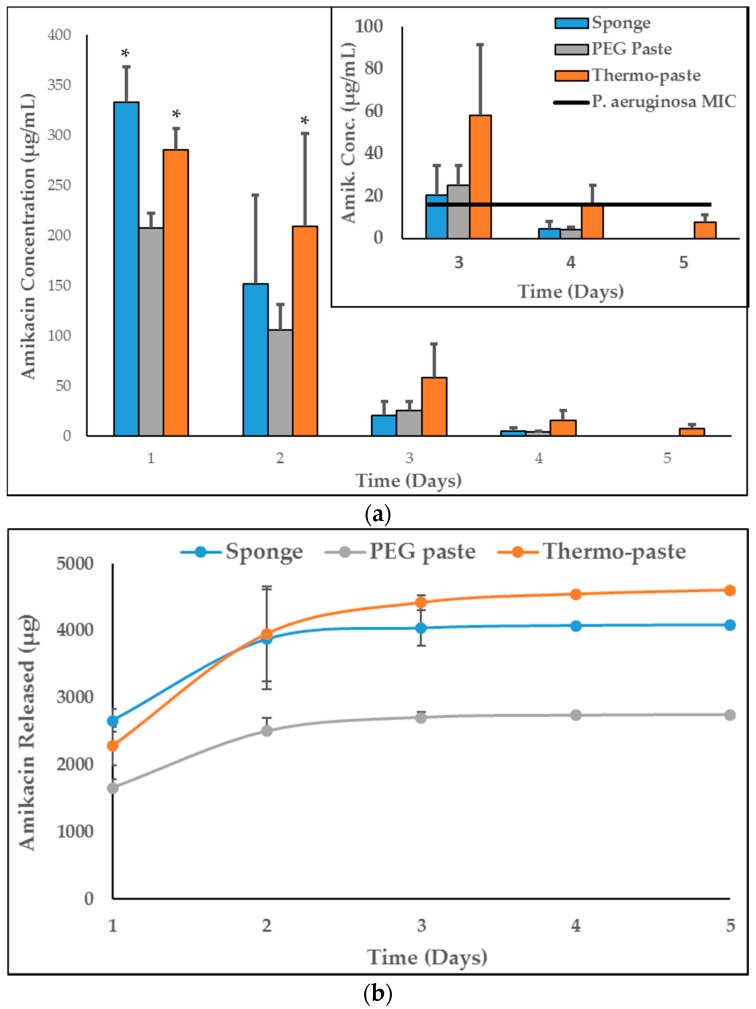
(**a**) Concentrations of amikacin in eluate samples, (**b**) cumulative amikacin released (n = 3). Data presented as mean ± STD. MIC values reported are lab generated values. * represents a significant difference compared to PEG paste (*p* < 0.05).

**Figure 5:**
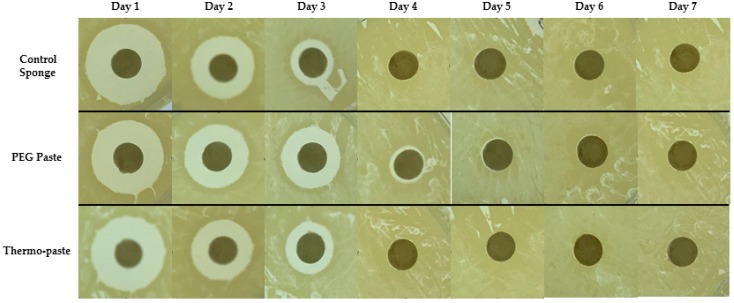
Representative ZOI images of eluates evaluated against *S. aureus*.

**Figure 6:**
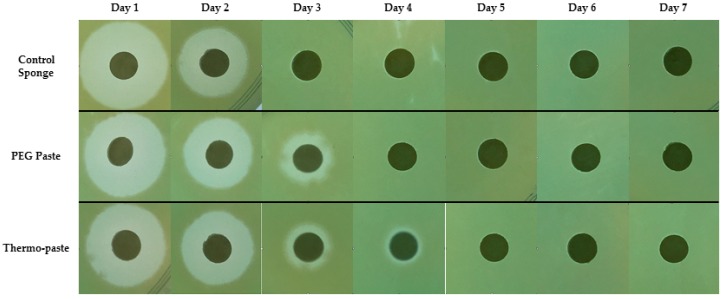
Representative ZOI images of eluates evaluated against *P. aeruginosa*.

The thermo-paste released both vancomycin and amikacin at infection-inhibiting levels for at least 72 h (Figure 3a and Figure 4a), and this was confirmed by zone of inhibition (ZOI) analysis (Table 1, Figure 5 and Figure 6). The vancomycin release kinetics of thermo-paste are comparable to poly(ethylene glycol) (PEG) paste with a cumulative release of 86.2 ± 14.3% vs. 91.9 ± 18.3% and detectable levels for 5 days (Figure 3b). Activity against *S. aureus* was sustained for 72 h based on ZOI analysis. Thermo-paste cumulatively released 46.0 ± 12.9% of amikacin vs. 27.4 ± 4.1% of PEG paste and maintained active concentrations against *P. aeruginosa* for 4 days (Figure 4b). Thermo-paste samples released amikacin at concentrations higher than PEG paste on Day 1 and were highest among all groups on Day 2. Detectable levels of antibiotics were not observed on Days 6 or 7 for any group. Extended elution profiles were observed for thermo-paste compared to chitosan sponge for both antibiotics.

### 2.3. Degradation

**Figure 7:**
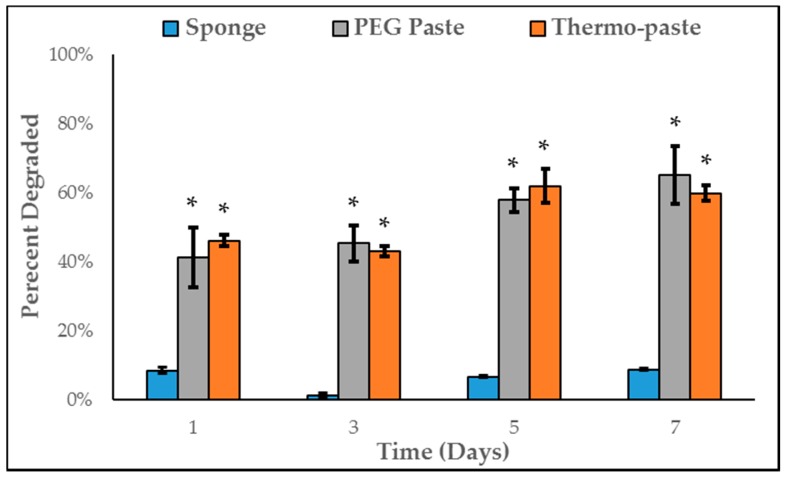
Enzymatic degradation of investigated systems (n = 3), data presented as mean ± STD; * represents a significant different compared to control sponge (*p* < 0.0001).

Thermo-paste and PEG paste demonstrated a higher degradation rate compared to the control sponge at each time point (*p* < 0.0001). After 24 h, thermo-paste and PEG paste samples were reduced to approximately 55% and 60% of their initial mass, respectively. No differences in degradation were observed between thermo-paste and PEG paste (Figure 7). The control sponge displayed minimal degradation, retaining approximately 100% of its original mass.

### 2.4. Injectability

**Figure 8:**
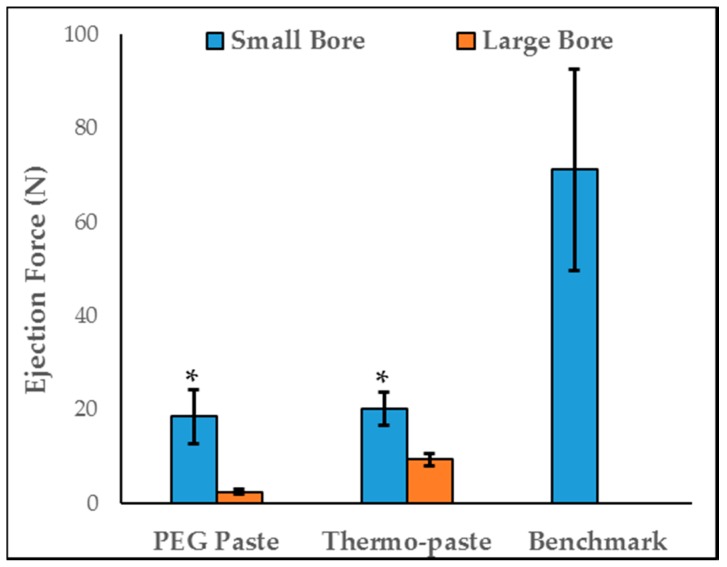
Injectability of paste samples (n = 3) from large diameter and small diameter syringes. Data are represented as mean ± STD; * represents a difference between bore diameters (*p* < 0.002) Benchmark established in the lab by sampling 10 personnel.

For both bore diameters, thermo-paste exhibited an ejection force similar to PEG paste, and both values were below the lab-generated benchmark (Figure 8). For thermo-paste and PEG paste, the small-bore syringe required more ejection force than the large-bore syringe (*p* < 0.002). Chitosan sponges are not injectable and were not evaluated.

### 2.5. Preclinical, Functional Model of Biocompatibility

**Figure 9:**
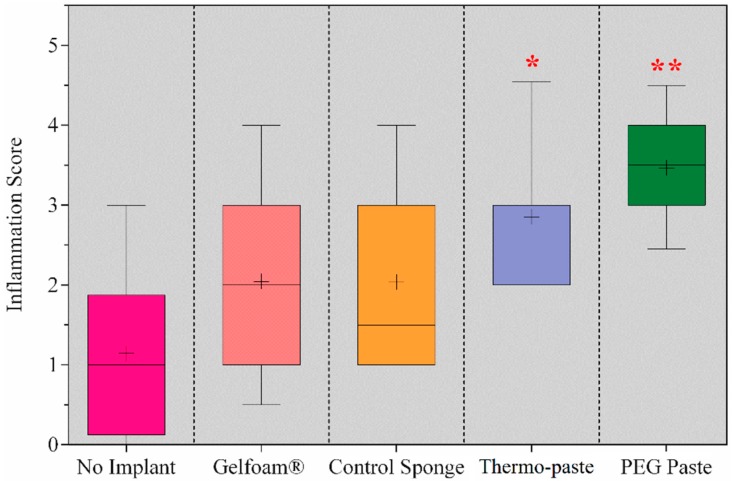
Histologic results presented as a 5–95 percentile box and whisker plot of average inflammation score with mean represented as (+); * represents a significant difference from no implant, Gelfoam^®^, and control sponge (* *p* ≤ 0.026, ** *p* < 0.0001).

Based on histological analysis of the degree of inflammation in the preclinical in vivo model, PEG paste elicited the highest inflammatory response. PEG paste and thermo-paste provoked an increased inflammatory response compared to control sponge, Gelfoam^®^ (Pfizer, Puurs, Belgium) gelatin sponge, and no implant groups. No differences were detected between thermo-paste and PEG paste, although PEG paste scored a higher inflammation score (3.5 vs. 2.9) (Figure 9).

## 3. Discussion

These experimental results indicate the potential of incorporating of β-GP into chitosan paste to improve the injectability and functionality of the system, while extending the release of amikacin. Clinician-selected antibiotics can be loaded in the system at the point of care to customize the system. Injectability and elution characteristics may enhance the use of the system as an adjunctive therapy to prevent infection in traumatic open wounds compared to sponge delivery systems. The extended release was accompanied by the improved activity of eluates against *P. aeruginosa*, without significantly affecting cellular compatibility or degradation. The combination of chitosan and β-GP demonstrates potential clinical use as an adjunctive infection prevention tool in complex open musculoskeletal trauma.

Thermo-paste cytocompatibility was similar to the chitosan sponge, which has been well documented as being biocompatible [15,25,26]. Ahmadi et al. reported on the response of fibroblast cells exposed to chitosan hydrogels composited with β-GP [22], concluding that a β-GP content of 10% (wt.) or less does not pose cytotoxic threats. Assad et al. investigated the same composite and hypothesized that the reduction in viability was due to a rapid release of β-GP increasing osmolality of the media, as reductions in viability were not observed after the media was changed [27]. Fibroblast cell viability was assessed using a static model, and this resulted in extended exposure to the materials. A dynamic fluid flow model or addition of media changes could be incorporated to more accurately represent a musculoskeletal wound and possibly improve in vitro cytocompatibility. Lowering β-GP content could improve thermo-paste cytocompatibility by attenuating the increase in osmolality. Increased viability of PEG paste compared to thermo-paste could be due to the addition of PEG, which has been shown to enhance the cytocompatibility of chitosan sponges [25], hydrogels [28,29], and chitosan pastes [20,21].

Elution profiles of both antibiotics showed a bolus release from all groups, but the extended release profile of paste delivery systems compared to chitosan sponges could be due to the slowed diffusion of antibiotics from the gel-like pastes. As the network becomes physically cross-linked and becomes more neutral, the release of macromolecular antibiotics may be slowed due to changes in the mesh size of the polymer network or changing interactions of charged antibiotic molecules within the system. The release kinetics of vancomycin from Pakzad et al.’s investigation of a composite of chitosan, gelatin, and β-GP are dissimilar to the current study, likely due to the incorporation of gelatin into the composite [30]. The authors noted that gelatin retards vancomycin elution, which could help explain the marked difference between initial burst releases, 3% vs. 64%. The amikacin and vancomycin elution kinetics from Berretta et al.’s investigation of a composite chitosan paste are similar to the results in the current study [21]. However, the delivery of active amikacin was extended from 72 h in Berretta et al. to 96 h, possibly due to electrostatic interactions between β-GP and amikacin. Thermo-paste eluted active concentrations of vancomycin and amikacin for at least 3 days, which would provide protection from invading pathogens during initial management of complex open musculoskeletal wounds [4]. Activity of eluted antibiotics was evaluated using a ZOI analysis which does not provide a quantitative reduction in bacterial burden. Further testing to expand upon these results will include determining bactericidal activity.

Differences in degradation between thermo-paste and chitosan controls could be due to increased surface area, acid content, or the presence of additives. An increased surface area allows for a larger portion of the delivery system to be exposed to lysozyme and enhances the degradation rate [31]. The degradation of paste systems compared to seemingly no degradation in lyophilized sponges supports this hypothesis. Rhodes et al. noted that the degradation rate of chitosan paste formulations accelerated with increased acidity [20]. The residual acid content of thermo-paste could be increasing the hydrolytic degradation. Once thermo-paste is hydrated, hydrogen ions on protonated amine groups dissociate and could promote dissolution of chitosan [9]. Additionally, composite chitosan scaffolds created from physical blending, such as the addition of PEG or β-GP, may be more susceptible to hydrolytic degradation than chitosan-only constructs due to interference with the crystallinity of chitosan [32,33]. Also, water may facilitate the breakdown of the weak polyelectrolyte complex formed between β-GP and chitosan. Despite not being completely degraded by the end of the study, thermo-paste may completely degrade in an in vivo setting. Stinner et al. investigated chitosan sponges in an infected in vivo model and demonstrated almost complete degradation over 42 h, but in this in vitro study, no degradation occurred over 168 h [19]. Accelerated degradation could be partially attributed to the presence of chronically activated macrophages that would promote acid-mediated hydrolysis [34].

MacDonald et al. reported the maximum ejection force of healthcare workers to be 79.5 N, which agrees with the lab-generated benchmark [35]. Rhodes et al. and Berretta et al. reported ejection force values of 150 N and 30 N, respectively, with similar chitosan composites [20,21]. The reduced ejection force of the thermo-paste could be due to the addition of β-GP. Thermo-paste has a low viscosity at room temperature (20 °C) and gels at body temperature (37 °C) which allows for the easy application and subsequent conversion to a more viscous state, in contrast with other previously developed chitosan pastes with high inherent viscosity and difficult injection [20,23]. Injectability facilitates enhanced wound coverage for complex musculoskeletal trauma and may improve the infection prevention capability by delivering antibiotics directly to the infection. 

VandeVord et al. observed no signs of pathological inflammation in a study of murine subjects subcutaneously implanted with lyophilized chitosan sponges for 12 weeks [14]. Increased levels of inflammation incited by the thermo-paste and PEG paste are likely due to the dissolution of the acidic components, decreasing the pH of tissue and bodily fluids immediately surrounding the implant. Thermo-paste may provoke a lower inflammatory response compared to PEG paste due to the neutralizing effect of β-GP and the incorporation of neutralized chitosan [36]. Inflammation scores of the thermo-paste were considered moderate and are comparable to similar in vivo biocompatibility studies of chitosan blended with PEG by Parker et al. and Rhodes et al. [20,25]. A study by Cui et al. observed a mild inflammatory response without the presence of a foreign body reaction in a similar composite of chitosan and β-GP [37]. The authors reported the superiority of the composite compared to unmodified chitosan membranes, which is similar to the results of the present study. Chitosan used in their study was not neutralized, and this could be a similar situation as comparing thermo-paste and PEG paste.

The functional and clinical efficacy of the thermo-paste are evaluated within the scope of open, complex musculoskeletal wounds which are resultant from high-energy trauma. Findings reveal thermo-paste is an injectable and degradable biomaterial capable of local antibiotic release. Preliminary results in this study support thermo-paste demonstrating potential for further development into an adjunctive tool for infection prevention in trauma care. Thermo-paste formulation should be further researched with extending antibiotic elution time and efficacy, as well as improving in vitro and in vivo biocompatibility. Future work will examine changes in viscosity and gelation to more completely characterize the basic material properties and gain additional insight into how the system works. Expanded in vivo studies will investigate thermo-paste in a model of complex extremity trauma for infection prevention efficacy.

## 4. Materials and Methods

### 4.1. Fabrication

Control chitosan sponges were prepared from chitosan (Chitopharm, Tromsø, Norway) with a molecular weight of 251 kDa and DDA of 82.5% by adapting methods outlined in Noel et al. [18]. Chitosan sponges were fabricated by dissolving 1% (*w*/*v*) chitosan in a 1% (*v*/*v*) acetic acid solution and stirring for 24 h (Table 2). After complete dissolution, the solution was frozen at −80 °C overnight and lyophilized for 3 days. Upon retrieval from the lyophilizer, the chitosan constructs were neutralized using 1.0 M NaOH and rinsed with copious deionized water to return the pH to 7. The sponges were again frozen at −80 °C overnight and lyophilized for 3 days. Control sponges were hydrated by allowing 120 mg of material to passively absorb aqueous solutions. A previously developed paste was fabricated from the methods in Berretta et al. for comparison [21]. PEG paste was produced by dissolving 1% (*w*/*v*) chitosan and 0.5% (*w*/*v*) poly(ethylene glycol) 8000 (PEG) (Sigma Aldrich, St. Louis, MO, USA) in a 0.85% (*v*/*v*) acetic acid solution (Table 2). This solution is stirred for 24 h, frozen at −80 °C, and lyophilized for 3 days. Paste was formed by grinding the lyophilized constructs into a particulate form and hydrating using 1 mL of PBS per 133 mg of dry powder.

Thermo-paste was fabricated by combining equal parts thermo-responsive chitosan composited with β-GP (EMD Millipore, Billerica, MS, USA) and control chitosan particulate. The thermo-responsive component was fabricated by dissolving 10% (*w*/*v*) β-GP and 2% (*w*/*v*) chitosan in a 0.5% (*v*/*v*) acetic acid solution (Table 2). After complete dissolution, the solution was frozen overnight at −80 °C, lyophilized for 3 days, and ground into particulate via a blade grinder. Prior to hydration, the composited and control chitosan components are combined in a 1:1 ratio by dry mass. Thermo-paste was hydrated at 120 mg of dry mass per 1 mL of PBS. The resultant paste is a temperature sensitive polymer with low viscosity at room temperature (20 °C). As temperature increases, the paste undergoes a gelation process at approximately 37 °C, resulting in a viscous paste.

### 4.2. Cytocompatibility

NIH3T3 cells (America Type Culture Collection, Manassas, VA, USA) were seeded in a 24-well plate at a density of 2 × 10^4^ cells/cm^2^ in 1 mL of cell culture media consisting of high glucose Dulbecco’s modified eagle medium (Fisher Scientific, Hampton, NH, USA), 10% (vol.) fetal bovine serum (Fisher Scientific, Hampton, NH, USA), and 100 µg/mL Normocin (InvivoGen, San Diego, CA, USA). After 24 h, media was aspirated and replaced with fresh culture media. The control sponge (120 mg), PEG paste (133 mg), and thermo-paste (120 mg) samples were hydrated with 1 mL of sterile phosphate buffered saline (PBS), added to porous cell culture inserts (n = 3, diameter 8 µm), inserted into the wells, and incubated at 37 °C for 72 h. The relative viability of NIH3T3 cells was quantified using a Cell Titer-Glo^®^ assay (Promega, Madison, WI, USA).

### 4.3. Antibiotic Elution and Activity

Samples (n = 3, PEG paste, control sponge, and thermo-paste) were hydrated with 1 mL of a 10 mg/mL vancomycin (Sigma Aldrich, St. Louis, MO, USA) and amikacin (Sigma Aldrich, St. Louis, MO, USA) solution. Samples were inserted into CellCrown™ inserts (Scaffdex, Finland) with dialysis membranes (diameter 100 µm) attached. Cell crowns were submerged in 8 mL of sterile PBS and gently shaken in an incubator at 37 °C for 7 days. Eluates were collected daily and frozen at −80 °C; the remaining solution was aspirated and completely refreshed with sterile PBS. Vancomycin in the eluate samples were measured using high performance liquid chromatography (Thermo Fischer Scientific, Waltham, MA, USA) interfaced with a UV/Vis spectrophotometer. Amikacin concentrations were measured using a spectrofluorometric protocol to detect aminoglycosides [38]. Eluate samples were diluted and reacted with a solution of acetylacetone, formaldehyde, and buffer composed of boric, acetic, and phosphoric acid adjusted to pH = 2.7. Absorbance values at 450 nm were measured with a Synergy^TM^ H1 plate reader (BioTek, Winooski, VT, USA).

Eluates were evaluated using a ZoI analysis. *Staphylococcus aureus* (ATCC 49230, America Type Culture Collection, Manassas, VA, USA) and *Pseudomonas aeruginosa* (ATCC 27317, America Type Culture Collection, Manassas, VA, USA) were chosen as representative bacterial strains because they are commonly found contaminating musculoskeletal wounds and capable of forming biofilm [39,40]. Tryptic soy agar (TSA) plates were inoculated with *S. aureus* or *P. aeruginosa*. Blank paper discs (diameter 6 mm) were hydrated with eluate samples (30 μL), placed on TSA plates, and incubated at 37 °C for 24 h. ImageJ software (National Institute of Health, Rockville, MD, USA) was used to measure the ZOI around each disc.

### 4.4. Degradation

The degradation of the thermo-paste was assessed as the reduction in dry mass over 7 days by the enzymatic degradation of lysozyme [15]. Initial mass of experimental samples was recorded, (n = 3/time point; control sponge, thermo-paste, PEG paste). Samples were hydrated with 2 mL of PBS, placed in porous (diameter 1.5 mm) hemisphere containers, and sealed using parafilm. The porous vessels containers were submerged in 40 mL of 1 mg/mL lysozyme solution (Lysozyme Type IV, MP Biomedicals, Santa Ana, CA, USA) and gently shaken in an incubator at 37 °C for the duration of the study. Every 48 h, the lysozyme solution was aspirated and completely replaced. On days 1, 3, 5, and 7, samples (n = 3/time point) were retrieved from the solution, dried overnight in a vacuum oven at 80 °C, and weighed. The percentage of initial mass remaining was calculated via Equation (1):

(1) $$\mathrm{Percent}\;\mathrm{of}\;\mathrm{Initial}\;\mathrm{Mass}\;\mathrm{Remaining}\;\left(\%\right)=\frac{\mathrm{Final}\;\mathrm{Sample}\;\mathrm{Mass}\;\left(\mathrm{mg}\right)}{\mathrm{Initial}\;\mathrm{Sample}\;\mathrm{Mass}\;\left(\mathrm{mg}\right)}\times \;100$$

### 4.5. Injectability

Injectability was assessed by ejecting 5 mL samples (n = 3) of PEG paste and thermo-paste from either a 10 mL syringe (bore diameter 2 mm, BD™, Franklin Lakes, NJ, USA) or 15 mL large bore syringe (bore diameter 7 mm, Qosina, Ronkonkoma, NY, USA). Syringes were fixed in an Instron Universal Testing Machine (Instron, Norwood, MA, USA) with a 5 kN load cell and programmed to depress the plunger at a rate of 1 mm/s. Injectability was quantified as the maximum ejection force required to eject the paste at 20 °C. The peak ejection force generated on 10 mL syringes by volunteers (n = 10) was measured using a load cell (Dillon Dynamometer, Liberty, MO, USA), averaged, and used as a benchmark to represent a relevant ejection force value.

### 4.6. Preclinical, Functional Model of Biocompatibility

Animal Care and Use Statement: Study protocols were approved by the University of Memphis IACUC protocol #0758. All appropriate measures were taken to minimize pain and discomfort.

**Figure 10:**
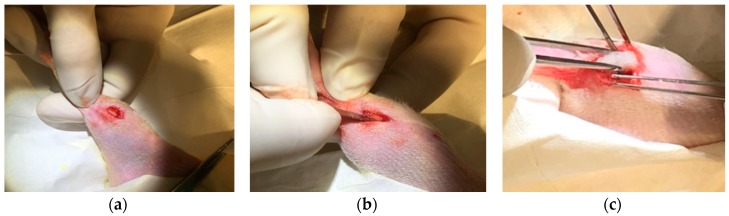
Wistar rats implanted with experimental systems to observe the inflammatory reaction: (**a**) 5 mm incision exposes the anterior tibia head; (**b**) bone defect was applied; (**c**) control sponge coupon inserted.

**Figure 11:**
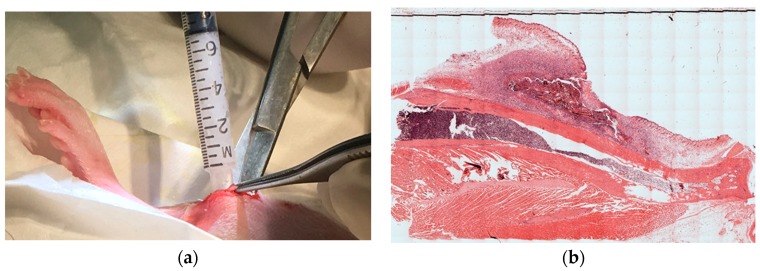
Wistar rats implanted with experimental systems to observe the inflammatory reaction: (**a**) paste samples injected; (**b**) H&E stained tissue section.

In vivo biocompatibility was assessed for control sponges (n = 3, 24 mg), Gelfoam^®^ gelatin sponges (n = 6, 24 mg), PEG paste (n = 6, 26 mg), and the thermo-paste (n = 6, 24 mg) with all samples being hydrated with 200 µL of sterile PBS. A control group was incorporated that received the same defect with the space remaining empty (n = 3). Male Wistar rats (Charles River North America, O’Fallon, MO, USA) were anesthetized using isoflurane inhalation. Right hind limbs were shaved and cleaned with betadine and isopropanol; an approximately 5 mm long incision was made superficially to the tibial tuberosity with a No. 10 scalpel blade, exposing the femoral epicondyle and proximal tibia head (Figure 10a). A defect was made with the scalpel to increase the hard tissue surface area exposed to the implant and as an identification marker for later histological processing (Figure 10b). Using surgical scissors, a muscular pouch was formed within the adductor muscular group in the medial lower thigh of the rats, and the experimental delivery systems were implanted within this pouch (Figure 10c and Figure 11a). Sutures sealed the muscular pouch, and the incision was closed with staples. The subjects were administered subcutaneous injections (0.1 mL) of Rimadyl 24 h and 48 h post-surgery for pain management and checked daily for redness and swelling. Rats were euthanized at 7 days, and the right hind limbs were excised and preserved in a 10% neutral formalin solution for a minimum of 14 days before histological processing. Tissue samples were decalcified and embedded in paraffin. Sections were cut perpendicular to the limb with an approximate thickness of 5 µm and stained with hematoxylin and eosin.

High-resolution montages of the stained tissue sections, fixed to histology slides, were created via a Nikon inverted microscope Eclipse TE300 operated by BIOQUANT OSTEO16 (BIOQUANT, Nashville, TN, USA) imaging software (Figure 11b). Qualitative histological analysis of the montages was performed by blinded reviewers (n = 9) evaluating the inflammation based on the Knodell histologic activity index used by Parker et al. [41,42]. The inflammation score range from 0 to 5: no inflammation scored a 0, very mild cellular aggregation scored a 1, moderately elevated cellular aggregation around the implant scored a 2, wide, dark rings of cellular aggregation around the implant scored a 3, thick layer of moderate cellular aggregation around the implant scored a 4, and severe cellular aggregation or the presence of a fibrous tissue capsule scored a 5.

### 4.7. Statistical Analysis

Statistical analysis was performed using GraphPad Prism 7.2 (GraphPad Software Incorporation, La Jolla, CA, USA). One-way analysis of variance (ANOVA) followed by Holm-Sidak’s post-hoc analysis was used to detect statistical differences among experimental groups in the cytocompatibility and injectability studies. Elution and degradation results were analyzed using a two-way ANOVA followed by Holm-Sidak’s post-hoc analysis to identify significant differences with time and experimental group as factors. In vivo functional compatibility results are non-parametric and analyzed using a Kruskal–Wallis one-way ANOVA followed by Dunn’s multiple comparisons test to identify statistical differences among experimental groups. *p* values < 0.05 were considered statistically significant.

## Figures and Tables

**Table 1:** Zone of inhibition (ZOI) diameters for eluate samples.

A. Staphylococcus aureus Zone of Inhibition Diameter (mm)							
Group	Antibiotic Eluate Time Point (day)						
	1	2	3	4	5	6	7
1. Control sponge	11.6	7.1	2.9	0	0	0	0
2. PEG paste	8.9	7.9	5.6	1.9	0	0	0
3. Thermo-paste	10.9	6.8	4.2	0	0	0	0
**B. *Pseudomonas aeruginosa* Zone of Inhibition Diameter (mm)**							
1. Control sponge	12.7	8.2	0	0	0	0	0
2. PEG paste	10.8	8.6	3.7	0	0	0	0
3. Thermo-paste	12.1	9.6	2.8	1.5	0	0	0

**Table 2:** Experimental chitosan group formulations.

Experimental Group		Chitosan (*w*/*v*)	Acetic Acid (*v*/*v*)	Additive (*w*/*v*)	pH
1. Control Chitosan		1%	1%	-	neutral
2. PEG Paste		1%	0.85%	0.5% PEG	acidic
3. Thermo-paste	50%	1%	1%	-	neutral
	50%	2%	0.5%	10% β-GP	acidic
